# The interplay between N6-methyladenosine and precancerous liver disease: molecular functions and mechanisms

**DOI:** 10.1007/s12672-023-00695-2

**Published:** 2023-05-25

**Authors:** Zhihua Lv, Ruoxi Ran, Yuting Yang, Meixian Xiang, Hanwen Su, Jingtao Huang

**Affiliations:** 1 Department of Clinical Laboratory, Institute of Translational Medcine, Renmin Hospital of Wuhan University, Wuhan, 430060 Hubei China; 2 Department of General Office, School of Stomatology, Wuhan University, Wuhan, China; 3 School of Pharmaceutical Sciences, South-Central University for Nationalities, Wuhan, China

**Keywords:** m^6^A, Liver disease, Molecular mechanisms, Carcinogenesis

## Abstract

N6-methyladenosine(m^6^A) is one of the most abundant modifications of mammalian cellular RNAs. m^6^A regulates various biological functions in epitranscriptomic ways, including RNA stability, decay, splicing, translation and nuclear export. Recent studies have indicated the growing importance of m^6^A modification in precancerous disease, influencing viral replication, immune escape, and carcinogenesis. Here, we review the role of m^6^A modification in HBV/HCV infection, NAFLD and liver fibrosis, and its function in liver disease pathogenesis. Our review will provide a new sight for the innovative treatment strategy for precancerous liver disease.

## Background

Liver cancer remains the seventh most frequently occurring cancer and the third leading cause of cancer death worldwide, with approximately 906,000 new cases and 830,000 deaths in 2020 [1, 2]. Hepatocellular carcinoma (HCC) is the major form of liver cancer and accounts for more than 80% cases [3]. Hepatitis B virus (HBV) and Hepatitis C virus(HCV) infection are the main risk factors for HCC development and account for 80% cases globally [4]. Besides, nonalcoholic fatty liver disease (NAFLD) and liver fibrosis are well-established risk factors for HCC [5]. Recently, epitranscriptomic modifications, especially RNA methylations, are known to result in changes in gene expression and virus life-cycle, and have been shown to trigger HCC [6].

**Fig. 1:**
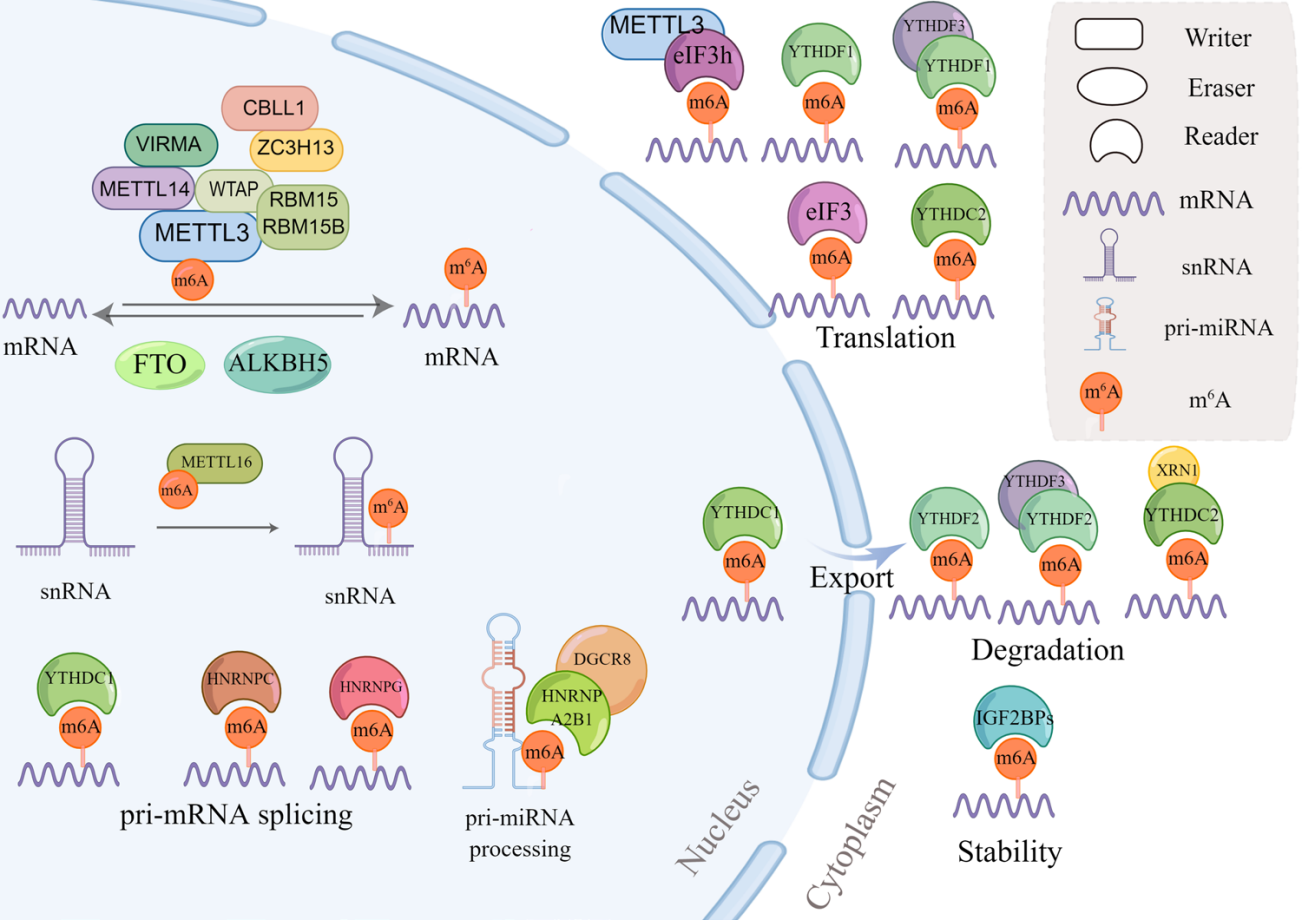
Molecular mechanism of m^6^A modification of RNAs. N6-methyladenosine methylation is a dynamic process that occurs in the nucleus. The m^6^A of RNAs is catalyzed by the writer complex, including METTL3, METTL14, WTAP, VIRMA, RBM15/15B, ZC3H13, CBLL1 and METTL16, and demethylated by erasers including FTO and ALKBH5. The m^6^A reader proteins, including YTHDF1/2/3, YTHDC1/2, IGF2BP1/2/3, HNRNPC/G/A2B1 and eIF3/3 h, recognize the m^6^A sites to modulate RNA stability, degradation, splicing, translation and nuclear export. RBM15/15B means RBM15 and RBM15B, YTHDF1/2/3 means YTHDF1,YTHDF2 and YTHDF3, YTHDC1/2 means YTHDC1 and YTHDC2, IGF2BP1/2/3 means IGF2BP1, IGF2BP2 and IGF2BP3, HNRNPC/G/A2B1 means HNRNPC, HNRNPG and HNRNPA2B1, eIF3/3 h means eIF3 and eIF3h. Draw by Figdraw

N6-methyladenosine(m^6^A) is the most abundant RNA methylation in eukaryotic RNA and widely occurs in mRNA and non-coding RNAs [ribosomal RNAs (rRNAs), tRNAs and circular RNAs (circRNAs)] [7, 8], and playing an essential role in regulating mammalian gene expression [7]. In mRNAs, most m^6^A sites are enriched in the 3′ untranslated region (UTR) and near stop codons [9, 10]. m^6^A most located in the consensus sequence RRACH (R = G or A and H = A, C, or U) [11]. The biological functions of m^6^A are mediated by corresponding enzymes, methyltransferases- “writers”, demethylases- “erasers” and “readers”. To date, an increasing number of m^6^A regulatory enzymes (writers, erasers and readers) have been discovered to involve in RNA stabilization, decay, splicing, translation, and nuclear export to influence liver disease development (Fig. 1).

### Dynamic regulation of m^6^A

#### Writers

As shown in Fig. 1 and Table 1, m^6^A methylation is a dynamic process regulated by m^6^A methyltransferase complexes, which consist of methyltransferase-like 3/14/16 (METTL3/14/16) [11, 12], Wilms’ tumor 1-associating protein (WTAP) [13], vir like m6A methyltransferase associated (VIRMA, also called KIAA1429) [14], RNA-binding motif protein 15 (RBM15) [15], Cbl photo oncogene like 1(CBLL1) [16] and zinc finger CCCH-type containing 13(ZC3H13) [17]. METTL3, as the first identified component, is the catalytic subunit that binds to S-adenosylmethionine (SAM) and catalyzes methyl transfer [18–20]. METTL14 serves as structural support for METTL3 and assists to binds to the target RNA; WTAP ensures nuclear speckle localization for METTL3-METTL14 heterodimer [13, 18, 19]. VIRMA is critical for recruiting core components METTL3/METTL14/WTAP to mediate preferential mRNA methylation in 3′UTR and near stop codon [14, 17]. RBM15 and its paralogue RBM15B interact with METTL3 and WTAP, and recruit them to specific RNA sites [15]. Other proteins, such as CBLL1 and ZC3H13, bridge WTAP to mRNA-binding factor [21–23]. Besides, METTL16 catalyzes m^6^A modification in U6 snRNA and plays an important role in pre-RNA splicing [24].

**Table 1:** Biological functions of m^6^A effectors

Effectors	Protein name	Location	Effect	References
Writers	METTL3	Nucleus and cytosol	Catalytic subunit of m^6^A; The cytoplasmic METTL3 enhances the translation of epigenetic mRNAs	[18–20]
	METTL14	Nucleus	Sructural support for METTL3 and assists to binds to the target RNA	[18, 19]
	WTAP	Nucleus	Ensure nuclear speckle localization for METTL3-METTL14	[13]
	VIRMA	Nucleus	Recruit METTL3/METTL14/WTAP to guide region-selective methylations	[17]
	RBM15/RBM15B	Nucleus	Interact with METTL3 and WTAP, and recruit them to specific RNA sites	[15]
	ZC3H13	Nucleus	Bind with RBM15/15B and link it to WTAP	[21, 22]
	CBLL1	Nucleus	Interact with WTAP to form complex	[23]
	METTL16	Nucleus	Catalyze m^6^A modification in U6 snRNA and plays an important role in pre-mRNA splicing regulation	[24]
Erasers	FTO	Nucleus	Catalyze m^6^A demethylation; catalyze m^6^A_m_ demethylation	[25, 26]
	ALKBH5	Nucleus	Catalyze m^6^A demethylation	[27]
Readers	YTHDF1	Cytosol	Recognize m^6^A-modified mRNA and promotes mRNA translation	[31]
	YTHDF2	Cytosol	Promote degradation of mRNA	[41]
	YTHDF3	Cytosol	Cooperate with YTHDF1 to promote protein synthesis, and affects methylated mRNA decay mediated through YTHDF2	[42]
	YTHDC1	Nucleus	Regulate mRNA splicing; Mediate nuclear export	[30, 43]
	YTHDC2	Nucleus and cytosol	Enhance the translation efficacy; Mediate mRNA degradation via recruiting XRN1 in cytosol	[33, 42, 43]
	HNRNPC	Nucleus	Pre-mRNA processing	[30]
	HNRNPG	Nucleus	mRNA alternative splicing	[36]
	HNRNPA2B1	Nucleus	Primary microRNA processing	[44]
	eIF3	Cytosol	Recognize m^6^A in the 5′UTR and initiates protein translation	[37]
	METTL3	Nucleus and cytosol	Bind with eIF3h to enhance translation	[45]
	IGF2BPs	Nucleus and cytosol	Recognize the consensus GG(m^6^A)C sequence and promote mRNA stability and translation	[38]

#### Erasers

m^6^A methylation can be reversed via demethylases that convert m^6^A into A, and two demethylases, fat mass and obesity-associated protein (FTO) and AlkB homolog 5 (ALKBH5), have been identified [25–27]. As a member of AlkB family, FTO was the first protein identified to catalyze m^6^A demethylation [25], and it can also demethylate m^6^A_m_ (N6,2′-O-dimethyladenosine) [28]. Both FTO and ALKBH5 are located in nuclear speckles and belong to Fe^2+^/α- ketoglutarate dependent dioxygenases enzyme family, which recognize m^6^A in mRNA [29].

#### Readers

m^6^A reader proteins recognize m^6^A modification and affect the fate of mRNAs to exert biological functions. To date, readers include the YT521-B homology (YTH) domain family proteins (YTHDF1/2/3) [30, 31], YTH domain containing proteins (YTHDC1/2) [32–34], heterogeneous nuclear ribonucleoprotein (HNRNPC, HNRNPG, and HNRNPA2B1) [30, 35, 36], eukaryotic translation initiation factor 3 (eIF3) [37], insulin-like growth factor 2 mRNA-binding proteins (IGF2BPs) [38]. The YTHDF family proteins have three similar paralogues, and with different effect on mRNA fate:YTHDF1 recognize m^6^A-modified mRNA and promotes mRNA translation [31], YTHDF2 selectively binding m^6^A-containing mRNA to promote degradation of mRNA [39], YTHDF3 cooperate with YTHDF1 to enhance protein synthesis, and affects methylated mRNA decay mediated via YTHDF2 [40]. YTHDC1 locates in nuclear and recruits pre-mRNA splicing factor SRSF3 to regulate mRNA splicing [32], and it also facilitates nuclear export of m^6^A-containing mRNA via interacting with SRSF3 and NXF1 [41]. YTHDC2 plays critical roles in mammalian germline by augmenting the translation efficiency of target methylated mRNA and diminishing their levels [33, 42]. Meanwhile, YTHDC2 regulate the stability of m^6^A-containing mRNAs by recruiting 5′–3′ exoribonuclease XRN1 [43]. m^6^A alters RNA structure to mediate the accessibility of RNA binding motifs, referred as ‘m^6^A-switch’ mechanism, which enhances binding of HNRNPC, an abundant nuclear RNA-binding protein responsible for pre-mRNA processing [30]. Besides, other RNA-binding proteins are also mediated via m^6^A-switch: HNRNPG is involved in mRNA alternative splicing, and HNRNPA2B1 is involved in primary microRNA processing [36, 44]. eIF3 is identified as an m^6^A reader, recognize m^6^A in the 5′UTR and initiates protein translation in a cap-independent manner [37]. METTL3 interacts with eIF3h and recognize the m^6^A sites close to the stop codon, and promote oncogenic mRNAs translation [45]. IGF2BPs, including IGF2BP1/2/3, recognize the consensus GG(m^6^A)C sequence and promote mRNA stability and translation [38].

### m^6^A modification in HBV infection

**Fig. 2:**
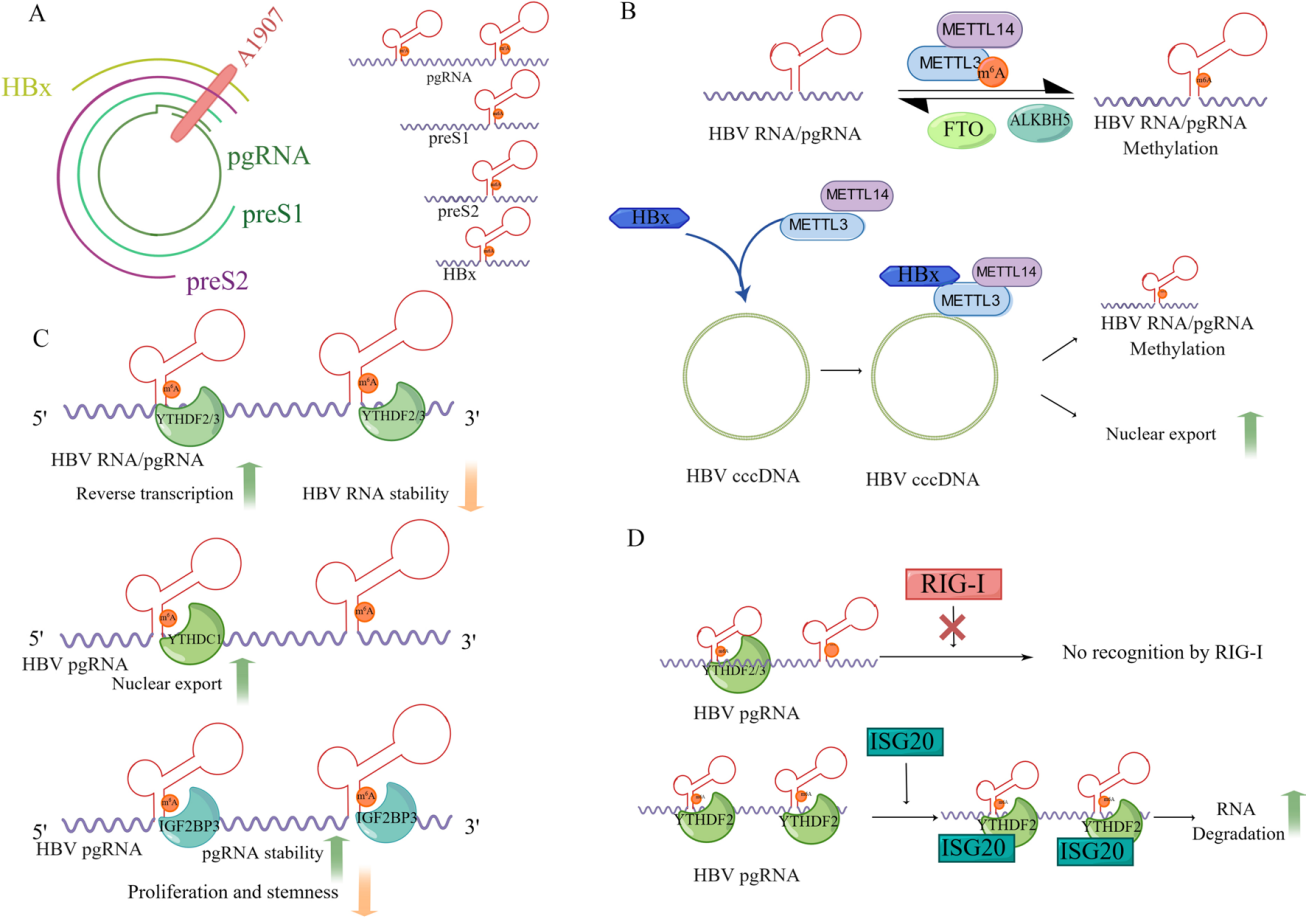
The functional role of m^6^A modification in HBV infection. **A** The m^6^A sites in HBV RNAs. HBV RNA is methylated at the single position (A1907) within the epsilon stem-loop region. The m^6^A site present in the 3′ terminus of all HBV RNAs and at both the 5′ and 3′ termini of the pgRNA. **B** The dynamic methylation of m^6^A in HBV RNAs. HBx recruits METTL3/METTL14 to the HBV cccDNA to catalyze m^6^A modification of HBV RNA/pgRNA, as well as promote nuclear import of m^6^A writers. **C** The m^6^A modification regulates HBV life cycle. YTHDF2/3 recognize the m^6^A site within 5′ epsilon stem-loop to increase the reverse transcription of pgRNAs and recognizes the m^6^A site within 3′ epsilon stem-loop to decrease the stability of HBV RNAs. Meanwhile, YTHDC1 promotes nuclear export of HBV pgRNAs and IGF2BP3 enhance the stability, reduce proliferation and stemness of HBV pgRNAs in an m^6^A-dependent manner. **D** The m^6^A modification regulates host immune responses. YTHDF2/3 hider RIG-I recognition of HBV RNAs to suppress immune response via interacting with m^6^A-modified HBV RNAs. ISG20 degrades HBV RNAs via interacting with YTHDF2 to recognize the m^6^A-modified HBV RNAs. YTHDF2/3 means YTHDF2 and YTHDF3. Draw by Figdraw

HBV, a member of the Hepadnaviridae family, contains a partially double-stranded DNA genome, which leads to chronic hepatitis and results in HCC development. Recent studies discovered that m^6^A modification could directly or indirectly regulate HBV replication (Fig. 2, Table 2).

**Table 2:** The regulators of m^6^A in different liver diseases

Disease	Regulator	Change	Sample source	Target	Mechanisms	Function	References
HBV infection	METTL3/14 YTHDF2/3	Downregulation	HepAD38 cells	5′ m^6^A site	m^6^A positively regulates reverse transcription	Lower HBV DNA levels	[46]
				3′ m^6^A site	m^6^A negatively regulates HBV RNA stability	Promote HBc/s protein expression and the half-life of pgRNA	[46]
	METTL3/14	Downregulation	HepAD38 cells	5′ m^6^A site	m^6^A promotes the viral RNA packaging efficiency; m^6^A facilitates the interaction with core proteins	Increase HBV-RNA and protein levels; Decrease packaged pgRNA, rcDNA and extracellular HBV-DNA	[47]
	METTL3/14	Recruitment by HBx protein onto cccDNA and PTEN	PHHs and HepG2 NTCP cells	HBV RNAs	METTL3/14 regulates stablility of HBV in a HBx-dependent manner; METTL3/14 increases nuclear import via HBx	Interact with HBx to carry out methylation activity on viral RNAs; Interact with HBx to stimulates their nuclear import	[48]
	YTHDC1	Downregulation	HepAD38 cells	5′ m^6^A site	YTHDC1 promotes nuclear export of m^6^A-mythlated HBV RNAs	Reduce viral DNA synthesis in the core particles, as well as the cccDNA levels	[50]
	FTO	Upregulation	HepG2.215 cell	HBV RNAs	FTO reduces the m^6^A levels of HBV RNAs	Increase cccDNA copy numbers of HBV	[49]
	IGF2BP3	Downregulation	HepG2.215 cell	pgRNAs	IGF2BP3 binds to pgRNA and enhances the stability of HBV-pgRNA	Suppress the proliferation, stemness, and tumorigenicity of pgRNAs	[51]
	METTL3/14 YTHDF2/3	Downregulation	HepG2 cells	5′ m^6^A site in pgRNAs	m^6^A suppresses retinoic acid–inducible gene I (RIG-I) sensing	Increase IFN-β synthesis and IRF-3 activation	[53]
	YTHDF2	Downregulation	HepG2 cells	pgRNAs	YTHDF2 interacts with ISG20 facilitates m^6^A modified HBV RNA degradation	Decrease the HBV RNA degradation	[54]
HCV infection	METTL3/14	Downregulation	Huh7 cells	m^6^A sites within the HCV E1 region	METTL3/14 regulates the production or release of infectious viral particles	Increase viral titer and protein	[56]
	FTO	Downregulation	Huh7 cells	m^6^A sites within the HCV E1 region	FTO regulates the production or release of infectious viral particles	Decrease viral titer and protein	[56]
	YTHDFs	Downregulation	Huh7 cells	m^6^A sites within the HCV E1 region	YTHDFs negatively regulate HCV particle production	Increase viral titer and protein	[56]
	WTAP	Downregulation	Huh7 cells	m^6^A sites within the HCV E1 region	WTAP negatively regulates HCV virion production in a METTL3-depent manner	Increase viral titer and protein	[59]
	YTHDC2	Downregulation	Huh7 cells	m^6^A sites within the HCV IRES element	YTHDC2 positively regulates HCV IRES-mediated translation via the m^6^A site at nt 331	Decreased the HCV IRES translation	[57]
	METTL3/14 YTHDF2/3	Downregulation	Huh7 cells	m^6^A sites at nucleotides 3′end genome of HCV RNA	m^6^A suppresses retinoic acid–inducible gene I (RIG-I) sensing	Increase IFN-β synthesis and IRF-3 activation	[53]
NAFLD	METTL3	Upregulation	mouse model of NAFLD	m^6^A sites at *Rubicon* mRNA	m^6^A increasing *Rubicon* expression and suppressing autophagic flux	Accelerate lipid droplets accumulation	[63]
	YTHDF1	Upregulation	mouse model of NAFLD	m^6^A sites at *Rubicon* mRNA	YTHDF1 binds to m^6^A-marked Rubicon mRNA and enhances its stability	Accelerate lipid droplets accumulation	[63]
	METTL3	Downregulation	obese leptin-deficient mice models	m^6^A sites at *DDIT4* mRNA	m^6^A induces DDIT4 improves NAFLD through downregulation of mTORC1 and NF-κB signaling pathways	Reduce hepatic steatosis,lipid staining and NAFLD activity inflammation scores	[64]
	METTL3	Downregulation	HepG2 cells and mice models	m^6^A sites at *PPaRα* mRNA	m^6^A increases *PPaRα* mRNA lifetime and expression in a YTHDF2-manner	Reduce lipid accumulation	[62]
	YTHDF2	Downregulation	HepG2 cells and mice models	m^6^A sites at *PPaRα* mRNA	YTHDF2 Mediates mRNA stability to increase *PPaRα* mRNA lifetime and expression	Reduce lipid accumulation	[62]
	YTHDC2	Downregulation	obese mice models and NAFLD patients	m^6^A sites at mRNA of lipogenic genes(*Srebf1*, *Fasn*, *Acaca*, *Scd* and *Gpam*)	YTHDC2 decreases mRNA stability and inhibits gene expression	Promote hepatic triglycerides accumulation	[65]
	METTL3/14	Upregulation	mouse model of NAFLD	m^6^A sites at ACLY and SCD1 mRNA	METTL3/14 increasse expression of ACLY and SCD1 in a m^6^A-dependment manner	Enhance lipid droplets accumulation	[66]
	FTO	Upregulation	mouse model of NAFLD	m6A sites at IL-17RA	FTO decreases m^6^A enrichment of IL-17RA	Facilitate liver injury and inflammation	[69]
	FTO	Downregulation	mouse model of NAFLD	3ʹUTR of *Srebf1*, *Fasn*, *Acaca* and *Scd* mRNAs	FTO regulates the expression of Srebf1, Fasn, Acaca and Scd in a m^6^A-dependment manner	Alleviate dexamethasone-induced fatty liver in mice	[70]
Hepatic fibrosis	METTL3	Upregulation	HSC-T6 cells	pri-miR-350	METTL3 binds with DGCR8 to regalate miR-350 systhesis in a m^6^A dependment manner	Promote liver fibrosis via ASIC1a	[73]
	WTAP	Downregulation	liver fibrosis mice models	–	–	Induce the development of liver fibrosis and promoting hepatic stellate cell activation	[74]
	METTL3	Upregulation	mouse model of liver fibrosis; Kupper cells	m^6^A sites at *MALAT1* mRNA	METTL3 enhances MALAT1 in a m^6^A-dependment manner, and acitivates MALAT1/PTBP1/USP8/TAK1 axis stimulated pyroptosis and inflammation of macrophages	Increase MALAT1 to promote liver fibrosis	[75]
	METTL3	Downregulation	mouse model of liver fibrosis	m^6^A sites at *Lats2* mRNA	Depleted METTL3 reduces m^6^A levels of Lats2 mRNA and suppressing its degradation;then upregulated Lats2 suppresses Hippo/YAP signaling pathway	Inhibit HSC activation and alleviate liver fibrosis	[76]
	YTHDF3	Upregulation	mouse model of liver fibrosis	m^6^A sites at *PRDX3* mRNA	YTHDF3 directly regulates the mRNA translation of PRDX3 in a m^6^A-dependent manner	Reduce hepatic profibrogenic and HSC activation, supress liver injury and fibrosis	[77]
	FTO	Downregulation	HSC-LX2 cells; mouse model of liver fibrosis	m^6^A sites at *BCEN1* mRNA	Downregulated FTO increases the expression of BECN1 in a m^6^A-dependment manner	Alleviate liver fibrosis by triggering HSC ferroptosis	[79]
	YTHDF1	Upregulation	HSC-LX2 cells; mouse model of liver fibrosis	m^6^A sites at *BCEN1* mRNA	YTHDF1 recognizes the m^6^A modification to stabilize the BECN1 mRNA	Alleviate liver fibrosis by triggering HSC ferroptosis	[79]
	ALKBH5	Downregulation	mouse model of liver fibrosis	m^6^A sites at *PTCH1* mRNA	ALKBH5 triggers PTCH1 activation in a m^6^A-dependent manner	Facilitate HSCs proliferation and migration	[80]
Liver regeneration	METTL3	Upregulation	mouse model of partial hepatectomy	–	Knocking out METTL3 suppresses hepatocyte cell cycle progression through SOCS6/STAT3 pathway	Suppress hepatocyte proliferation	[81]
	METTL14	Upregulation	mouse model of partial hepatectomy	–	METTL14 deficiency leads to arrestion of G1 phase and excessive endoplasmic reticulum (ER) stress after PHx	Loss of METTL14 impairs liver regeneration	[83, 84]
Liver development	METTL3	–	Hepatic Mettl3 knockout neonatal mice model	m^6^A sites at *Hnf4a* mRNA	Deletion of METTL3 reduce the half-life of *Hnf4a* mRNA in an IGF2BP1-dependent manner	Loss of METTL3 triggers apoptosis, steatosis and fibrosis, resulting in severe liver damage in newborn mice	[82]
Alpha-1 antitrypsin deficiency	METTL14	Upregulation	mouse model of AATD	m^6^A sites at *CHOP* mRNA	METT14 inhibits CHOP expression by promoting CHOP mRNA decay in an m^6^A-dependent manner	Alleviate the liver damage induced by excessive ER stress	[85]

Imam and colleagues discovered that there is a conserved m^6^A consensus motif (A1907) located within the epsilon stem-loop region [46]. The m^6^A site is present at the 3′ terminus of all HBV RNAs and at both the 5′ and 3′ termini of the pgRNA [46]. Depleting of METTL3/METTL14 or YTHDF2/YTHDF3 results in increased expression of HBcAg and HBsAg; conversely, knocking down of FTO/ALKBH5 reduces the expression of HBV proteins [46]. Besides, researchers discovered that the m^6^A modification at the 5′ stem-loop plays positive roles for reverses transcription of pgRNA while m^6^A at 3′ stem-loop lower the stability of HBV RNAs and viral protein production [46]. Recently, another pivotal role of m^6^A modification in HBV life cycle was discovered[47]. The methyltransferases (METTL3/METTL14) influenced the encapsidation of HBV pgRNA, and depleting of methyltransferases increase HBV-RNA and protein levels but decrease levels of packaged pgRNA, rcDNA and extracellular HBV-DNA [47]. The m^6^A modification of 5′ epsilon stem-loop region increased the viral RNA packaging efficiency and facilitated the interaction with core proteins, though m^6^A modification of 3′ stem was unessential for viral encapsidation [47]. A recent study showed that HBV X (HBx) protein binds to m^6^A methyltransferases and mediated m^6^A modification, which reduces the stability of HBV RNAs and promotes the nuclear import of METTL3/ METTL14 [48]. Recently, a targeted RNA demethylation by SunTag system (TRADES) was established [49]. This system is based on CRISPR/Cas and recruits RNA demethylase to demethylate the target m^6^A site [49]. Researchers targeted HBV RNA via TRADES and lead to ~ threefold increase of HBV cccDNA copy numbers [49]. Decreased m^6^A levels of pgRNA reduce HBV DNA levels and promote viral protein production, including the HBx, which may promote the nuclear import of METTL3/14 and reverse the demethylase function. Another study uncovered that YTHDC1 and FMRP recognize m^6^A-methylated HBV RNAs and facilitate their nuclear export [50]. Besides, depletion of YTHDC1 or FMRP could reduce viral DNA synthesis in the core particles, as well as the cccDNA levels [50]. Another study demonstrated that IGF2BP3, an m^6^A reader, binds to pgRNA and enhances the stability of HBV-pgRNA, meanwhile, pgRNA up-regulates IGF2BP3 expression at the posttranscriptional level via miR-let-7e-5p [51]. Silencing IGF2BP3 could significantly abrogate the proliferation, stemness and tumorigenicity of pgRNA [51].

Recent years, m^6^A modifications were confirmed to regulate innate immunity and antiviral response [52]. It has recently been established that m^6^A modification site (A1907) of HBV pgRNA 5′ stem-loop was involved in regulating the innate immune response [53]. Mutant of m6A modification site (A1907C) enhances retinoic acid–inducible gene I (RIG-I) sensing, stimulating IRF-3 activation and IFN signal. Besides, depletion of METTL3 and METTL14, as well as YTHDF2/3, promotes HBV RNA recognition by RIG-I, and leads to an increase in interferon production [53]. Notably, YTHDF2/3 hider RIG-I recognition of HBV RNAs via interacting with m^6^A-modified HBV RNAs, resulting in inhibiting RIG-I mediated immune response [53]. Furthermore, the HBV transcripts can be bound and degraded by the IFN-induced interferon-stimulated gene 20 (ISG20) [54]. Moreover, ISG20 degrades HBV transcripts via interacting with YTHDF2 to recognize the m6A modification of HBV RNAs [53]. The mutant HBV RNAs, which lack m^6^A methylation at both termini of HBV RNAs, were resistant to ISG20-mediated suppression [54]. PTEN, a tumor suppressor protein, mediates dephosphorylation of IRF-3 at Ser-97 to induce IRF-3 nuclear import and IFN synthesis [55]. Kim et all found that HBV infection suppressed IFN signaling via increasing the m^6^A modification of PTEN mRNA which resulted in RNA instability [55].

### m^6^A modification in HCV infection

HCV, belonging to the *Flaviviridae* family, is a positive single-stranded RNA virus and cause chronic Hepatitis C, which is another major etiology factor of HCC. The RNA genomes of *Flaviviridae* family, including HCV, dengue, Zika, yellow fever, and West Nile virus, were modified by m^6^A in conserved regions [56].

**Fig. 3:**
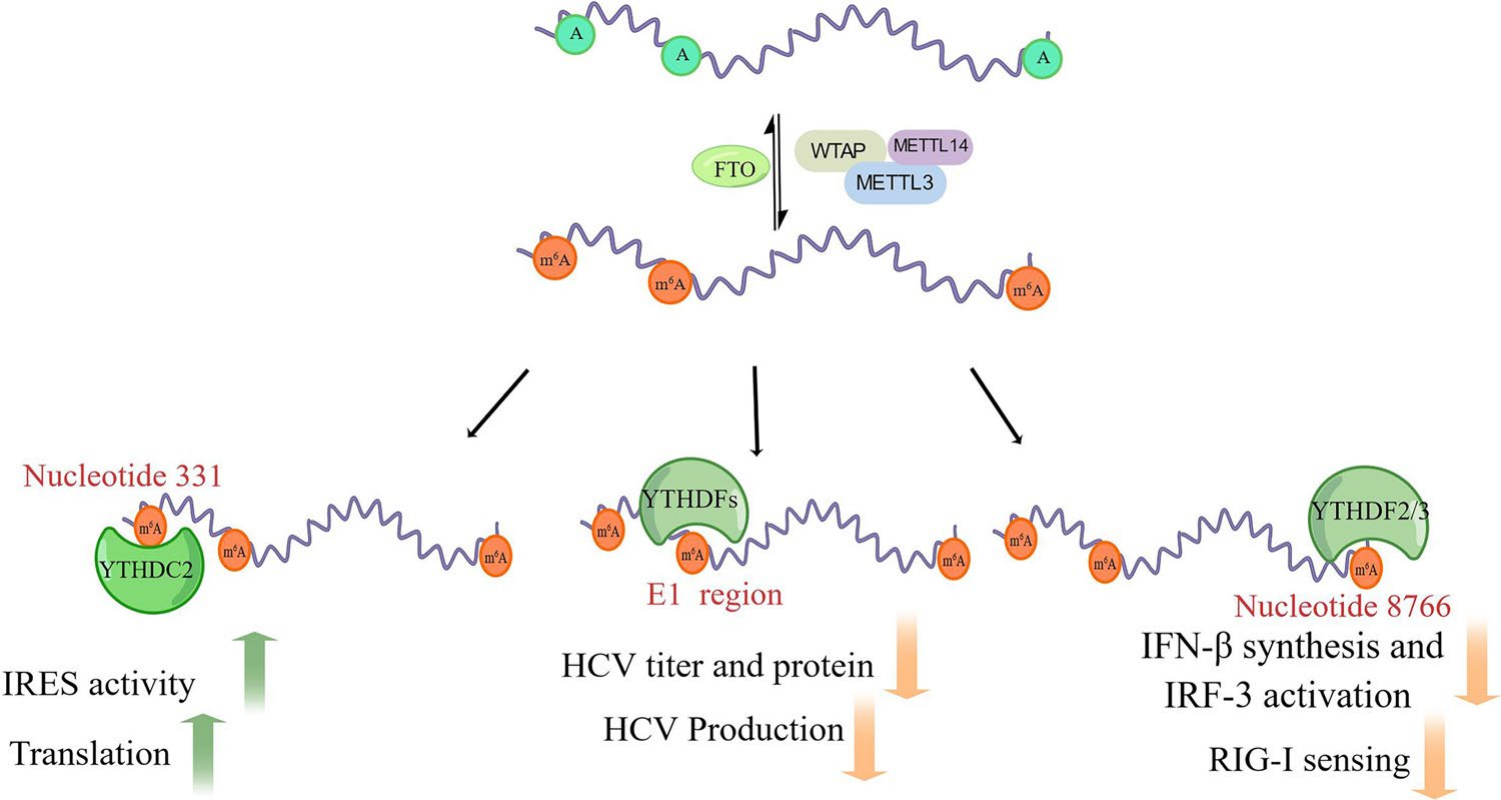
The functional role of m^6^A modification in HCV infection. The HCV genome contains several m^6^A-modified regions (~ 19 regions), including E1 region, IRES element and 3′ end genome region. YTHDC2 recognizes the m^6^A modification in IRES element influence HCV translation initiation. YTHDFs recognize the m^6^A modification in HCV E1 gene to regulate viral RNA packaging. The m^6^A site at 3′ end HCV genome (~ 100 bp upstream of PAMP), recognized by YTHDF2/3, affects the function of PAMP by reducing its sensitivity to RIG-I. YTHDF2/3 means YTHDF2 and YTHDF3. Draw by Figdraw

Harnessing methyl-RNA immunoprecipitation (MeRIP) sequencing, Gokhale and colleagues identified that HCV RNA is modified by m^6^A and there are ~ 19 m^6^A peaks at the HCV RNA genome (Fig. 3) [56]. Remarkably, m^6^A modification in HCV RNA has been observed to negatively regulate extracellular viral RNA levels and viral particle production, without any impact on HCV translation or RNA replication [56]. Notably, all three YTHDF proteins bind to m^6^A-methylated HCV RNA, leading to the relocation of HCV RNA to lipid droplets to suppress HCV particle production [56]. Besides, there are several m^6^A modification sites within HCV RNA genome, and each m^6^A modification may have different functions depending on its location. For example, the m^6^A modification in HCV E1 gene regulates viral RNA packaging via recognizing by YTHDF proteins [56], the m^6^A modification in the internal ribosome entry site (IRES) influences translation [57]. A recent study discovered that there is an m^6^A site at HCV nucleotide(nt) 331, located about 10 nt upstream of initiation codon (AUG) [57]. YTHDC2 recognizes m^6^A methylation at nt 331 to regulate viral life cycle by inducing the HCV IRES translation activity [57]. Similar to HBV infection, depleting of METTL3 and METTL14,as well as YTHDF2 and YTHDF3, significantly increased IFN-β synthesis and IRF-3 phosphorylation in HCV infection cell models [53]. The pathogen-associated molecular patterns (PAMPs), nucleotides 8872–9616 in HCV genome, recognized by RIG-I affect IFN-β level and activation of IRF-3 [58]. There is no m^6^A modification in the HCV PAMP RNA, but an m^6^A modification site (nucleotide 8766) was identified at ~ 100 bp upstream of PAMP RNA. Via constructing A8766C-mutated models, authors discovered that m^6^A modification of HCV 8766 nucleotide affects the function of PAMP by reducing their sensitivity to RIG-I [53].

Another study spotlighted on the mechanisms of m^6^A modifications in HCV infection [59]. Depletion of both WTAP and METTL3 + 14 led to decreased m^6^A levels on HCV RNA and m^6^A modified transcript during HCV infection [59]. METTL3 directly binds with HCV RNA in a WTAP-dependent manner to reduce the production of infectious viral particles. Like METTL3 + 14, WTAP declines infections of HCV virion in a METTL3-dependent manner but does not alter HCV RNA replication in the cytoplasm [59].

### m^6^A modification in NAFLD

**Fig. 4:**
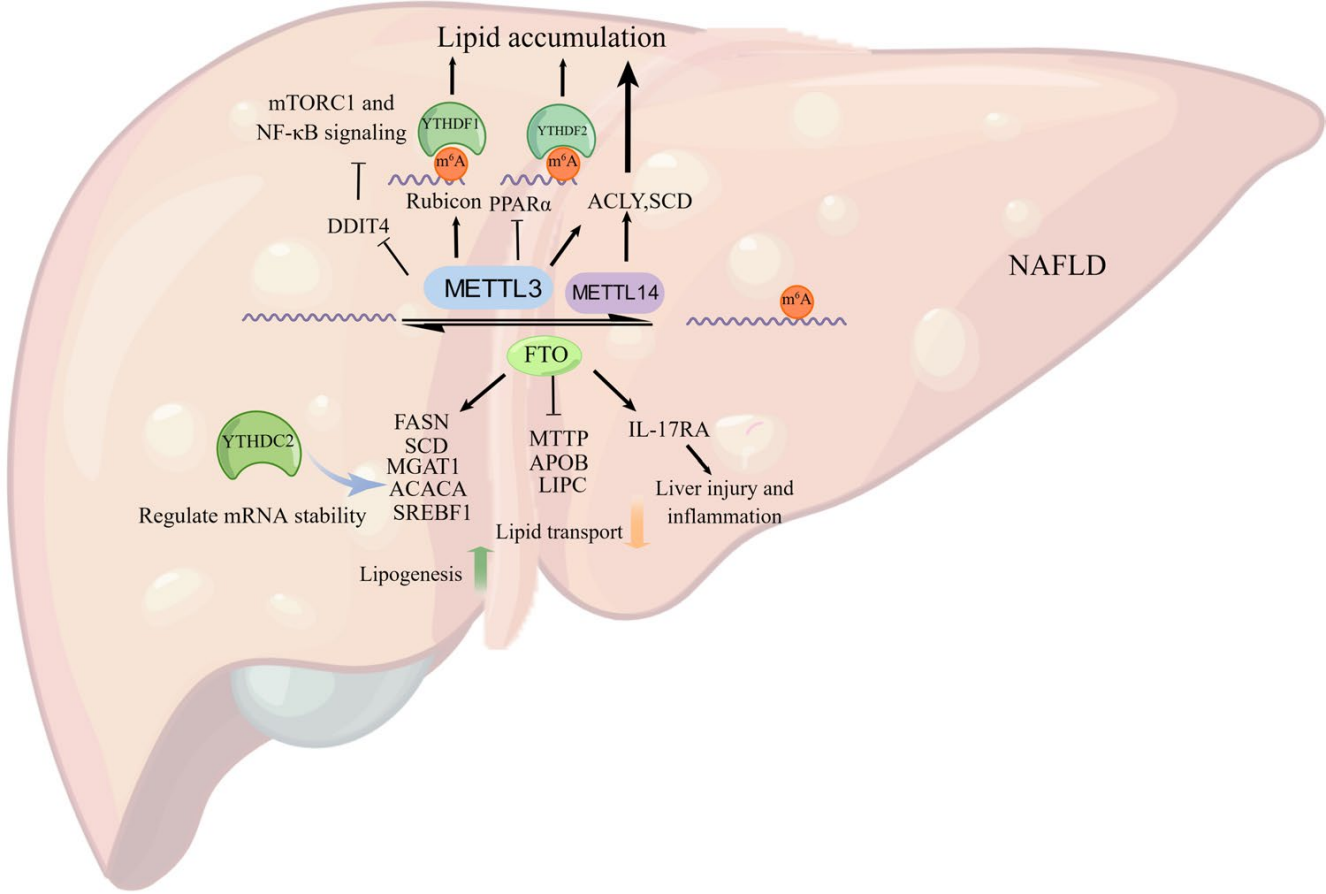
The functional role of m^6^A modification in NAFLD. In NAFLD patients, the lipogenesis, metabolism-associated and lipid transport genes are regulated by m^6^A effectors in an m^6^A-dependent manner to affect NAFLD development. Draw by Figdraw

Nonalcoholic fatty liver disease (NAFLD), which is rapidly emerging among children and adolescents, has become another significant risk factor foe HCC [60]. NAFLD, characterized by excessive triglyceride accumulation and steatosis in hepatocytes, develops to nonalcoholic steatohepatitis (NASH) with hepatic inflammation and hepatocytic injury, ultimately leading to hepatic cirrhosis and/or HCC [61]. Recent studies have investigated the m^6^A methylation plays a crucial role in hepatic lipid metabolism and the development of NAFLD (Fig. 4) [62].

The m^6^A writers and readers also participate in the regulation of NAFLD development. In a high fat diet (HFD)-induced mouse model of NAFLD, the m^6^A level and the methyltransferases METTL3 were found to be significantly upregulated [63]. METTL3-mediated m^6^A modification suppressed autophagic flux by targeting *Rubicon* mRNA and upregulating its expression to accelerate lipid droplet accumulation [63]. Meanwhile, YTHDF1 enhances the stability of *Rubicon* mRNA by binding to it, leading to inhibition of autophagic flux and accelerating lipid droplet accumulation [63]. In another study, loss of METTL3 leads to a reduction in the activity of mTOR and NF-κB signaling pathways, resulting from the stabilization of m^6^A-mediated DNA Damage Inducible Transcript 4 (DDIT4) mRNA in macrophages, which regulated metabolic reprogramming in NAFLD and obesity [64]. Besides, the circadian clock regulates hepatic lipid metabolism in an m^6^A-dependent manner. Knockdown of METTL3 decreased *PPaRα* m^6^A abundance, and prolonged *PPaRα* mRNA lifetime via recognized by YTHDF2, which reduced lipid accumulation [62]. Recently, YTHDC2 was identified as a regulator of hepatic lipogenesis through recognition of m^6^A sites and reduce mRNA stability of lipogenesis genes [65]. Authors observed a downregulation of YTHDC2 in both obese mice models and NAFLD patients. They also found that the overexpression of YTHDC2 suppresses lipogenic genes (*SREBF1*, *FASN*, *ACACA*, *SCD* and *GPAM*) mRNA stability to reduce cellular TG contents [65]. Yang and colleagues established a new NAFLD model and discovered lipid metabolism was regulated by ACLY and SCD via m^6^A-modified manner [66]. Overexpression of METTL3 or METTL14 significantly enhanced lipid droplet accumulation by elevating the m^6^A levels of ACLY and SCD gene transcripts, finally resulting in up-regulated expression [66].

Apart from being an m^6^A demethylase, FTO is also a crucial regulator in glucose and lipid metabolism, and it may regulate NAFLD in an m^6^A-dependent manner [67]. Overexpressed FTO enhances lipid accumulation by increasing the expression of metabolism-associated genes (*FASN*, *SCD*, and *MGAT1*), while simultaneously downregulating the expression of lipid transport genes (*MTTP*, *APOB*, and *LIPC*) [68]. Meanwhile, mutant FTO (R136A) with no demethylation activity did not have these functions [68]. Using corticosterone (CORT)-induced fatty liver models, authors discovered that the m^6^A level of mRNA was decreased and FTO was increased in liver. Besides, interleukin-17A (IL-17A), one isoform of IL-17 family cytokines, plays pivotal roles in the pathogenesis of NASH, which regulated by m^6^A modulator FTO [69]. Researchers revealed decreased IL-17RA m^6^A levels and increased IL-17RA protein levels in the progression of HCC [69]. In murine NAFLD models, FTO facilitates liver injury and inflammation via decreased m^6^A enrichment of IL-17RA mRNA [69]. Meanwhile, the levels of m^6^A on the lipogenic genes (*SREBF1*, *FASN*, *ACACA* and *SCD)*, were significantly decreased under dexamethasone combined with oleic acid (OA/ DEX) treatment and resulted in the upregulation of proteins [70]. Notably, meclofenamic acid (MA), an FTO inhibitor, could alleviate OA/DEX–induced m^6^A hypomethylation on the lipogenic genes. Besides, knockdown of FTO alleviates DEX-induced fatty liver in mice as well [70].

### m^6^A modification in hepatic fibrosis

**Fig. 5:**
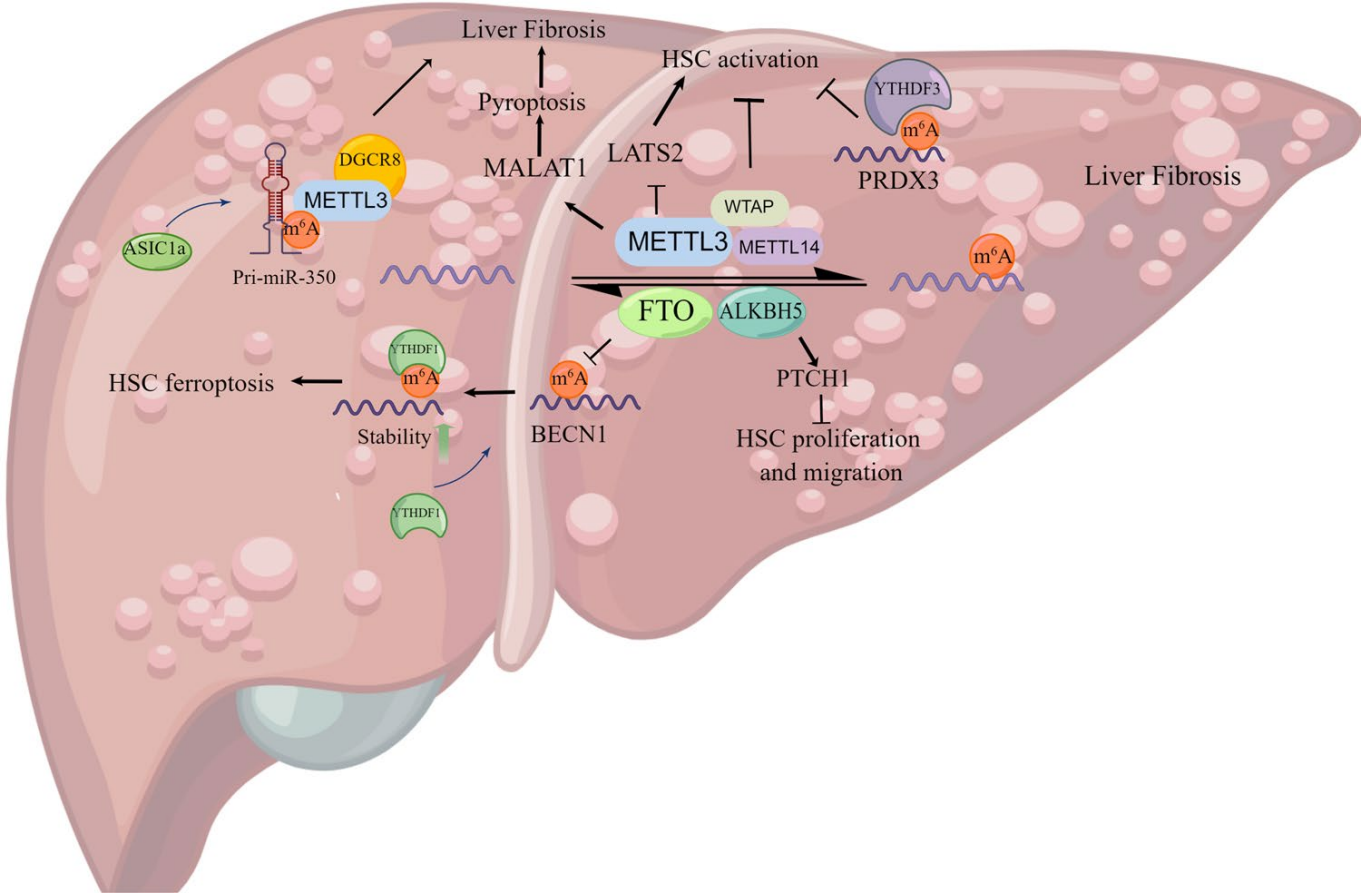
The functional role of m^6^A modification in liver fibrosis. The activation, proliferation, migration and ferroptosis ability of HSCs are regulated by m^6^A effectors in an m^6^A-dependent manner to influence liver fibrosis process. Draw by Figdraw

Hepatic fibrosis increases the risk of HCC, and ~ 4% of cirrhosis cases develop to HCC per year [71]. As a critical post-transcriptional modification, m^6^A methylation plays a vital role in liver fibrosis progress (Fig. 5).

Recent study discovered that m^6^A modification not only involved in the progression and reversal of hepatic fibrosis, but also participated the dynamic regulation of fibrosis [72]. For example, the m^6^A level of CCR2 (a chemokine receptor) is markedly elevated in the progression of liver fibrosis, while decreased in the reversal of liver fibrosis, indicating its potential as a therapeutic target [72]. *ASIC1a* was discovered upregulated in liver fibrosis, mediated m^6^A regulation of miR-350 mature to participate in liver fibrosis via PI3K/AKT and ERK pathways [73]. Mechanistically, METTL3 was associated with *ASIC1a* promoting liver fibrosis and subsequently regulated the DGCR8-mediated synthesis of miR-350 by m^6^A modification [73]. In the liver fibrosis mice, the m^6^A methylation abundance was significantly decreased as well as the expressions of WTAP, ALKBH5 and YTHDF1, and the decreased expression of WTAP has been shown to induce the development of liver fibrosis and promote hepatic stellate cell (HSC) activation [74]. Likewise, in liver fibrosis models, METTL3 and MALAT1 were upregulated in Kupffer cells and macrophages cells. The METTL3/MALAT1/PTBP1/USP8/TAK1 axis stimulated pyroptosis and inflammation of macrophages, leading to the aggravation of liver fibrosis [75]. Overexpressed METTL3 increased MALAT1 expression through m^6^A modification, and then MALAT1 directly bound with PTBP1 to down-regulated USP8, which resulted in reduced ubiquitination of TAK1 [75].

The activation of HSCs is a key process of liver fibrosis, and HSC-specific knockout of METTL3 inhibits HSC activation and significantly alleviates liver fibrosis [76]. Via reducing m^6^A levels of *LATS2* mRNA and suppressing its degradation, upregulated LATS2 promoted the phosphorylation of the downstream transcription factor YAP. This process led to the downregulation of pro-fibrotic gene expression and affected the Hippo/YAP signaling pathway to mitigate liver fibrosis progress [76]. YTHDF3 directly regulated the mRNA translation of PRDX3 in an m^6^A-dependent manner to alleviate liver fibrosis [77]. PRDX3 acted as a master regulator of mitochondrial oxidative stress and suppressed HSC activation via the (ROS)/TGF-β1/Smad2/3 pathway [77]. Besides, the activation of HSCs was attenuated under dihydroartemisinin (DHA) treatment via the ferroptosis pathway in an m^6^A-mediated manner [78]. The DHA downregulated FTO and boosted m^6^A abundance of BECN1 mRNA [79]. Subsequently, YTHDF1 recognized the m^6^A modification to stabilize the BECN1 mRNA, thus leading to autophagy activation and inducing HSC ferroptosis [79]. ALKBH5 regulated liver fibrosis by affecting the stability of *PTCH1* mRNA in an m^6^A-mediated manner [80].

### m^6^A modification in other liver diseases

Besides, m^6^A modification has been uncovered associated with liver regeneration. Researchers observed that the m^6^A level and METTL3 were significantly upregulated in the 2/3 partial hepatectomy (PHx) mice. By constructing liver-specific METTL3 knock-out mouse model, researchers discovered hepatocyte proliferation was suppressed, and the hepatocyte cell cycle progression was delayed through the SOCS6/STAT3 pathway [81]. Moreover, perinatal deletion of METTL3 triggers apoptosis, steatosis and fibrosis, resulting in severe liver damage and ultimately culminating in postnatal death within 7 weeks [82]. Deletion of METTL3 inducing a significant decrease of m^6^A deposition on transcription factor, *Hnf4a*, via reducing the half-life of *Hnf4a* mRNA in an IGF2BP1-dependent manner [82]. Smilarly, loss of METTL14 has been shown to impair liver regeneration after PHx, with extensive parenchymal necrosis [83, 84]. The METTL14-deficented hepatocytes are arrested in the G1 phase of cell cycle, as well as inducing excessive endoplasmic reticulum (ER) stress [83]. Alpha-1 antitrypsin deficiency (AATD) was a prevalent inherited liver disease characterized by misfolded protein accumulation in the endoplasmic reticulum (ER), ultimately leading to inflammation, fibrosis,cirrhosis and HCC. However, it is noteworthy that the majority of patients do not develop liver toxicity [85]. Wei and his collegues discovered that misfolded proteins in AATD are involved in suppressing the ubiquitination of METTL14 via an interaction with HRD1. Consequently, the expression level of METTL14 is up-regulated, leading to the alleviation of ER-stress induced liver damage via promoting CHOP mRNA decay in an m^6^A-dependent manner [85].

## Conclusion and perspectives

RNA methylation, especially m^6^A modification, has been discovered that play vital roles in various biological functions [34, 37, 37, 39]. RNA biology, including splicing, stability, editing, translational and degradation, is involved in liver diseases in an m^6^A-dependent manner [46, 56, 73]. In this review, we have delineated the dynamic m^6^A modification process, and described the functional effects of methylation in various liver diseases, including HBV infection, HCV infection, NAFLD and liver fibrosis. It has become clear that the m^6^A modification plays an outsized role in premalignant disease of HCC and participated in the progress to HCC.

Recent studies have demonstrated that the HCV RNA, as well as the RNA transcripts of HBV, are administrated by m^6^A modification to regulate viral life cycle, virus particle production and the process of pathogenesis [46, 51, 53, 54, 56]. Here, we discussed the functions of m^6^A modification in HBV and HCV infections. Notably, m^6^A modifications regulate the HBV and HCV life cycles, packaging and nuclear import in complex ways depending on their location in the HBV RNAs and HCV genome. Additionaly, it has been observed that m^6^A methylation plays a critical role in regulating host RNAs during HBV and HCV infections, thereby affecting the immune escape of the virus and contribute to viral persistence and chronicity [48]. Eventually, the regulation of m^6^A modification during HBV and HCV infections has a significant impact on the development of liver disease and promotes liver carcinogenesis.

NAFLD is another important risk factor for HCC, and NAFLD-related HCC accounts for 1% to 38% of the HCC burden in different countries/regions [60]. The m^6^A demethyltransferase, FTO, is involved in the lipid metabolism. The downstream molecules (*FASN*, *ACACA*, *SCD* et al.), regulated by FTO in the m^6^A-dependent manner, are implicated in lipogenesis, lipid transport to participate in the process of NAFLD. Meanwhile, METTL3 and METTL14 regulate the m^6^A sites at mRNAs of various lipid metabolism genes to promote lipid accumulation, which further leads to the progression of NAFLD and HCC [66].

Liver fibrosis is an important premalignant disease leading to HCC, and effective treatment remains absent. The activation of HSCs promotes the development of liver fibrosis, which is regulated by m^6^A modification in complex ways. In this review, we detailed that *MALAT1*, *LATS2*, *PRDX3*, *PTCH1* and *BECN1* genes affect the HSCs activation, proliferation, migration and ferroptosis in an m^6^A-dependent manner, which regulates the progress of liver fibrosis.

In liver regeneration, m^6^A methyltransferase plays an important function as well. In the PTx mice models, knocking out METTL3 results in suppressed hepatocyte proliferation delayed cell cycle as well as METTL14. Noteably, loss of METTL14 in PTx mice leads to excessive ER stress [83], and the misfolded proteins in AATD patients upregulates METTL14 to protect against ER stress-induced liver damage [85]. These results indicate that m^6^A modification is critical for maintaining endoplasmic reticulum homeostasis.

Thus, m^6^A modification is a dynamic and reversible process that plays diverse functions in premalignant liver disease. Understanding the mechanisms and clinical implications of m^6^A modification is crucial for preventing or intervening in the occurrence and development of liver diseases and HCC. Further research on the methylation mechanism and its role in liver disease will provide valuable insights into the therapy strategy for the treatment of liver diseases and HCC. Overall, comprehensively understanding the m^6^A modification and its clinical implications can transform the field of liver disease and HCC research.

## Data Availability

Not applicable.

## Abbreviations

m^6^A: N6-methyladenosine
HBV: Hepatitis B virus
HCV: Hepatitis C virus
NAFLD: Nonalcoholic fatty liver disease
HCC: Hepatocellular carcinoma
METTL3: Methyltransferase-like 3
WTAP: Wilms’ tumor 1-associating protein
VIRMA: Vir like m6A methyltransferase associated
RBM15: RNA-binding motif protein 15
CBLL1: Cbl photo oncogene like 1
ZC3H13: Zinc finger CCCH-type containing 13
SAM: S-adenosylmethionine
UTR: Untranslated region
FTO: Fat mass and obesity-associated protein
ALKBH5: AlkB homolog 5
m^6^A_m_: N6,2′-O-dimethyladenosine
YTHDFs: YT521-B homology domain family proteins
YTHDCs: YTH domain containing proteins
HNRNPC: Heterogeneous nuclear ribonucleoprotein C
eIF3: Eukaryotic translation initiation factor 3
IGF2BPs: Insulin-like growth factor 2 mRNA-binding proteins
SRSF3: Serine and arginine rich splicing factor 3
NXF1: Nuclear RNA export factor 1
XRN1: 5′–3′ Exoribonuclease 1
pgRNA: Pregenomic RNA
HBcAg: Hepatitis B core antigen
HBsAg: Hepatitis B surface antigen
rcDNA: Relaxed circular DNA
RIG-I: Retinoic acid–inducible gene I
IRF-3: Interferon regulatory factor 3
IFN: Interferon
ISG20: Interferon-stimulated gene 20
PAMP: Pathogen-associated molecular patterns
DDIT4: Damage inducible transcript 4
PPaRa: Peroxisome proliferator activated receptor alpha
SREBF1: Sterol regulatory element binding transcription factor 1
FASN: Fatty acid synthase
ACACA: Acetyl-CoA carboxylase alpha
SCD: Stearoyl-CoA desaturase
GPAM: Glycerol-3-phosphate acyltransferase, mitochondrial
ACLY: ATP citrate lyase
MGAT1: Alpha-1,3-mannosyl-glycoprotein 2-beta-N-acetylglucosaminyltransferase
MTTP: Microsomal triglyceride transfer protein
APOB: Apolipoprotein B
LIPC: Lipase C, hepatic type
ASIC1a: Acid-sensing ion channel 1a
CCR2: C–C motif chemokine receptor 2
DGCR8: DGCR8 microprocessor complex subunit
MALAT1: Metastasis associated lung adenocarcinoma transcript 1
HSC: Hepatic stellate cell
LATS2: Large tumor suppressor kinase 2
PRDX3: Peroxiredoxin 3
PTCH1: Patched 1
BECN1: Beclin 1
PHx: Partial hepatectomy
ER: Endoplasmic reticulum
AATD: Alpha-1 antitrypsin deficiency
